# Optical Coherence Tomography and Optical Coherence Tomography Angiography-Derived Retinal Microvascular and Neuroretinal Measures in Adults With Ischemic Stroke and Carotid Artery Stenosis: A Systematic Review and Meta-Analysis

**DOI:** 10.7759/cureus.113478

**Published:** 2026-07-27

**Authors:** Fidaa Mohammed Eltigani, Khadega Siddeg Abakr, Sara Barakat Mohammed Saeed, Shaimaa Tagelsir Mustafa Abdelsalam, Khairiah Abker Mohamed Yasan, Abla Yousif, Ahmed Abdalla

**Affiliations:** 1 Ophthalmology, Sudan Medical Specialization Board, Khartoum, SDN; 2 Ophthalmology, Saudi Aramco, Al-Hasa, SAU; 3 Accident and Emergency, Sudanese Consortium for Surgical Development, Khartoum, SDN; 4 Ophthalmology, Aljanah Medical Clinics, Ar Rass, SAU; 5 Medical Department, Cavan General Hospital, Cavan, IRL

**Keywords:** carotid artery stenosis, ischemic stroke, optical coherence tomography, optical coherence tomography angiography, retinal nerve fiber layer

## Abstract

Adults with ischemic stroke or carotid artery stenosis may exhibit retinal microvascular and neuroretinal changes that can be assessed non-invasively using optical coherence tomography (OCT) and optical coherence tomography angiography (OCTA). This systematic review and meta-analysis evaluated OCT- and OCTA-derived retinal microvascular and neuroretinal measures in these populations. Eligible original studies were synthesized narratively. Quantitative synthesis was limited to continuous outcomes with sufficiently similar anatomical and imaging definitions and extractable means, standard deviations, and sample sizes for both cerebrovascular disease and comparator groups. Deep retinal vessel density showed a moderate pooled standardized difference, with lower average measurements in the cerebrovascular disease groups and moderate heterogeneity detected. The retinal nerve fiber layer (RNFL)/peripapillary retinal nerve fiber layer (pRNFL) and ganglion cell complex (GCC)/ganglion cell layer (GCL) analyses showed small pooled standardized differences, with no heterogeneity detected; however, each analysis included only three comparisons. Superficial and radial peripapillary or optic disc vessel density measurements were also lower on average, but substantial to high between-study heterogeneity limited uniform interpretation. The foveal avascular zone (FAZ) area analysis included only two comparisons and was considered exploratory. Separate small observational studies reported associations involving FAZ morphology, retinal vascular orientation, cerebral hemodynamics, and post-revascularization perfusion changes; however, these findings represent distinct hypothesis-generating signals and do not establish OCTA as a validated surrogate for cerebral perfusion or demonstrate a unified clinical pathway. Overall, OCT- and OCTA-derived retinal measures may help characterize group-level differences in selected retinal microvascular and neuroretinal measurements among adults with ischemic stroke or carotid artery stenosis, but these measures have not been validated for diagnostic or prognostic use. The review was not prospectively registered. Most included studies had moderate risk-of-bias concerns, while two were rated as moderate-to-high risk. The evidence was further limited by predominantly observational designs, variation in imaging protocols and comparator structures, and sparse quantitative evidence for several outcomes. Further prospective studies using standardized OCT and OCTA acquisition, segmentation, and reporting methods are required before these measures can support routine clinical interpretation or risk stratification.

## Introduction and background

Stroke remains a major cause of death and long-term disability worldwide, with ischemic stroke accounting for a substantial proportion of the global cerebrovascular disease burden [1]. Carotid artery stenosis is a related but clinically distinct vascular condition. Depending on its severity and clinical presentation, carotid artery stenosis may be symptomatic or asymptomatic and may contribute to cerebral hypoperfusion, embolic events, and future ischemic risk [2]. Ischemic stroke represents established cerebral ischemic injury, whereas carotid artery stenosis is an upstream large-vessel disorder that may affect cerebral and ocular perfusion. These populations were included within the same review because both may be associated with retinal vascular and neuroretinal changes detectable using retinal imaging, although they should not be regarded as a single homogeneous disease group. Cerebrovascular injury may also involve small-vessel mechanisms, including endothelial dysfunction and impaired autoregulation [3]; however, these mechanisms provide background context and were not evaluated as separate exposures in this review.

The retina is an optically accessible neural and vascular tissue that shares selected anatomical, developmental, and neurovascular features with the central nervous system [4]. These similarities provide a biologically plausible basis for investigating retinal changes in cerebrovascular disease, but they do not make retinal and cerebral tissues functionally interchangeable. The retinal circulation has its own layered vascular organization, local physiological characteristics, and autoregulatory behavior, and retinal measurements may be influenced by ocular features, vascular comorbidities, and imaging methodology. Retinal imaging is therefore best considered a potential source of indirect and complementary information about selected aspects of cerebrovascular disease rather than a direct measurement of cerebral perfusion or neural injury [5].

Optical coherence tomography (OCT) and optical coherence tomography angiography (OCTA) have expanded retinal assessment beyond conventional fundus photography. OCT provides high-resolution structural measurements of retinal and choroidal tissues. OCTA detects motion contrast generated by circulating blood cells and produces flow-signal-derived measures, such as vessel density or perfusion density, rather than direct measurements of absolute blood flow [6]. The measures evaluated in this review therefore represent structurally and technically distinct parameters. Retinal nerve fiber layer (RNFL), ganglion cell complex (GCC) or ganglion cell layer (GCL), and macular and choroidal thickness are structural measurements, whereas superficial and deep capillary plexus density, radial peripapillary or optic disc vessel density, and foveal avascular zone (FAZ) parameters are OCTA-derived vascular measurements. These parameters should not be interpreted as interchangeable because OCTA measurements are indirect and may vary according to the imaging device, scan protocol, image processing, and segmentation method.

Previous studies using conventional retinal imaging have supported a broader association between retinal vascular features and cerebrovascular outcomes. Yatsuya et al. [7] reported that conventional retinal microvascular abnormalities were associated with the risk of lacunar stroke in the Atherosclerosis Risk in Communities Study. McGeechan et al. [8] found that retinal vessel caliber, particularly wider venular caliber, was associated with incident stroke events in a systematic review and individual-participant meta-analysis. Girach et al. [9] subsequently summarized evidence concerning retinal imaging and stroke-risk assessment and reported associations between several retinal features and future stroke risk. These studies provide background support for investigating retinal changes in cerebrovascular disease; however, conventional fundus-derived abnormalities and prognostic associations are not equivalent to the OCT- and OCTA-derived group differences evaluated in adults with established ischemic stroke or carotid artery stenosis.

Despite the growing literature, important uncertainty remains. Earlier evidence largely examined conventional fundus-derived signs, broader retinal-cerebrovascular associations, or future stroke risk, whereas OCT- and OCTA-derived retinal microvascular and neuroretinal measures in adults with established ischemic stroke or carotid artery stenosis remain less clearly synthesized. Existing studies differ in their disease populations, comparator structures, imaging devices, scan protocols, segmentation methods, and outcome definitions. These differences limit cross-study comparability, quantitative pooling, and the generalizability of pooled estimates rather than the interpretation of each study individually. A focused synthesis is therefore needed to determine whether predefined retinal imaging measures differ across the relevant disease-specific comparisons and which findings are sufficiently aligned for quantitative analysis.

The aim of this systematic review and meta-analysis was to evaluate predefined OCT- and OCTA-derived retinal microvascular and neuroretinal imaging measures in adults with ischemic stroke and carotid artery stenosis, treating these as related but clinically distinct populations. Specifically, the review evaluated superficial capillary plexus density, deep capillary plexus density, radial peripapillary or optic disc vessel density, FAZ parameters, RNFL or peripapillary retinal nerve fiber layer (pRNFL) thickness, and GCC or GCL thickness in relation to the relevant comparison structures. These included healthy or non-stroke controls, contralateral eyes, ischemic stroke subtype groups, and pre- and post-revascularization assessments, where applicable. Independent continuous group comparisons with sufficiently aligned outcome definitions and extractable means, standard deviations, and sample sizes were considered for quantitative synthesis. Correlation analyses, subtype comparisons, contralateral-eye comparisons, and pre-post findings that were unsuitable for pooling were synthesized narratively.

## Review

Methods

This systematic review and meta-analysis was reported in accordance with the Preferred Reporting Items for Systematic Reviews and Meta-Analyses (PRISMA) 2020 statement [10]. The review evaluated OCT- and OCTA-derived retinal microvascular and neuroretinal imaging measures in adults with ischemic stroke and carotid artery stenosis. The review was not prospectively registered. To support transparency, the eligibility criteria, record management procedures, screening process, data extraction approach, risk-of-bias assessment, and synthesis methods are reported below. Published review articles, including systematic reviews and meta-analyses, were used only for reference list screening and citation tracking and were not included in either the narrative or quantitative synthesis. The review question was structured using the PICO (population, intervention or exposure, comparator, and outcomes) framework before study selection, as presented in Table 1.

**Table 1:** PICO framework for the systematic review and meta-analysis. PICO: population, intervention or exposure, comparator, and outcomes; OCT: optical coherence tomography; OCTA: optical coherence tomography angiography; FAZ: foveal avascular zone; RNFL: retinal nerve fiber layer; pRNFL: peripapillary retinal nerve fiber layer; GCC: ganglion cell complex; GCL: ganglion cell layer.

PICO element	Definition used in this review
Population	Adults aged 18 years or older with ischemic stroke confirmed according to the clinical or neuroimaging criteria used by the source study, or with symptomatic or asymptomatic carotid artery stenosis as defined by the source study. One study of broader carotid atherosclerotic burden was retained only as secondary narrative evidence.
Exposure or index condition	Ischemic stroke or carotid artery disease evaluated using OCT, OCTA, or both, with the two clinical populations treated as related but distinct.
Comparator	Healthy or non-stroke controls for independent group comparisons; contralateral eyes, ischemic stroke subtype groups, or pre-post revascularization comparisons for narrative synthesis where applicable.
Outcomes	OCT- or OCTA-derived retinal and choroidal imaging measures, including superficial capillary plexus vessel density, deep capillary plexus vessel density, radial peripapillary or optic disc vessel density, FAZ area or morphology, RNFL or pRNFL thickness, GCC or GCL thickness, macular thickness, and choroidal thickness.

A systematic literature search was conducted in PubMed/MEDLINE, Scopus, Web of Science, and Embase using English language search terms. The search covered records published from database inception to July 3, 2026, with no publication date restrictions applied during the database search. The search strategy combined terms related to ischemic stroke, carotid artery stenosis, retinal imaging, OCT, OCTA, retinal vessel density, FAZ, RNFL, GCC, GCL, and choroidal measures. Reference lists of relevant reviews, meta-analyses, and eligible original articles were also screened manually to identify additional primary studies. Reviews were used only for citation tracking and were not included as primary studies. The database-specific search strategies are presented in Table 2.

**Table 2:** Search strategy used to identify eligible studies. Search strings were adapted to the syntax of each database. OCT: optical coherence tomography; OCTA: optical coherence tomography angiography; FAZ: foveal avascular zone; RNFL: retinal nerve fiber layer; GCC: ganglion cell complex; GCL: ganglion cell layer.

Source	Search strategy
PubMed/MEDLINE	("ischemic stroke" OR "ischaemic stroke" OR stroke OR "cerebral infarction" OR "carotid artery stenosis" OR "internal carotid artery stenosis" OR "carotid stenosis") AND ("optical coherence tomography angiography" OR OCTA OR "optical coherence tomography" OR OCT) AND (retina OR retinal OR "retinal vessel density" OR "vessel density" OR "perfusion density" OR "foveal avascular zone" OR FAZ OR "retinal nerve fiber layer" OR RNFL OR "ganglion cell complex" OR GCC OR "ganglion cell layer" OR GCL OR choroid OR choroidal)
Scopus	TITLE-ABS-KEY(("ischemic stroke" OR "ischaemic stroke" OR "cerebral infarction" OR "carotid artery stenosis" OR "internal carotid artery stenosis" OR "carotid stenosis") AND ("optical coherence tomography angiography" OR OCTA OR "optical coherence tomography" OR OCT) AND (retina OR retinal OR "vessel density" OR "foveal avascular zone" OR FAZ OR RNFL OR GCC OR GCL OR choroid OR choroidal))
Web of Science	TS=(("ischemic stroke" OR "ischaemic stroke" OR "cerebral infarction" OR "carotid artery stenosis" OR "internal carotid artery stenosis" OR "carotid stenosis") AND ("optical coherence tomography angiography" OR OCTA OR "optical coherence tomography" OR OCT) AND (retina OR retinal OR "vessel density" OR "foveal avascular zone" OR FAZ OR RNFL OR GCC OR GCL OR choroid OR choroidal))
Embase	('ischemic stroke' OR 'ischaemic stroke' OR stroke OR 'cerebral infarction' OR 'carotid artery stenosis' OR 'internal carotid artery stenosis' OR 'carotid stenosis') AND ('optical coherence tomography angiography' OR octa OR 'optical coherence tomography' OR oct) AND (retina OR retinal OR 'vessel density' OR 'foveal avascular zone' OR faz OR rnfl OR gcc OR gcl OR choroid OR choroidal)
Manual citation searching	Reference lists of relevant reviews, meta-analyses, and eligible original studies were screened to identify additional original human studies.

Studies were eligible if they included adults aged 18 years or older with ischemic stroke or carotid artery stenosis and reported relevant OCT- or OCTA-derived retinal, choroidal, microvascular, or neuroretinal outcomes. Ischemic stroke was accepted when diagnosed according to the clinical or neuroimaging criteria used by the source study, including acute, established, or subtype-defined ischemic stroke populations. Carotid artery stenosis studies could include symptomatic or asymptomatic disease. No uniform restriction was imposed on stroke timing or minimum stenosis severity beyond the definitions used by the original investigators; these characteristics were extracted and considered when judging clinical comparability. One study that evaluated broader carotid atherosclerotic burden using carotid intima-media thickness and plaque categories was retained only as secondary narrative evidence because it directly examined relevant OCTA measures; it did not contribute to the principal pooled analyses. Eligible designs included original human cross-sectional comparative, case-control, cohort, prospective or retrospective observational, and non-randomized pre-post studies. Studies were eligible for quantitative synthesis when they reported continuous data from independent comparative groups, with extractable means, standard deviations, and sample sizes, and when the populations, comparator structure, anatomical region, and outcome definitions were sufficiently similar to represent the same broad retinal domain. Device platform, scan dimensions, segmentation boundaries, and image-processing methods were considered when judging comparability but were not assumed to be interchangeable. Studies reporting odds ratios, regression estimates, correlation coefficients, subtype comparisons, contralateral-eye comparisons, or pre-post data without an eligible independent control group were retained for narrative synthesis when relevant to the review question.

Studies were excluded if they were review articles, including systematic reviews and meta-analyses, editorials, commentaries, protocols, case reports, conference abstracts without sufficient data, letters without eligible original data, animal studies, laboratory studies, cadaveric studies, or pediatric studies. Studies were also excluded if they did not include an eligible cerebrovascular population or relevant subgroup, did not use OCT or OCTA, did not report relevant retinal or choroidal outcomes, focused only on unrelated ocular disease without separate cerebrovascular data, represented duplicate or overlapping populations without additional relevant information, had insufficient data for either quantitative or narrative synthesis, or if the full text remained unavailable after reasonable retrieval attempts. Methodological quality and risk of bias were not eligibility criteria and were not used as reasons for full-text exclusion. Risk-of-bias assessment was performed only after final study inclusion and was used to guide interpretation of the evidence.

All retrieved records were imported into Zotero (Corporation for Digital Scholarship, Vienna, VA). Potential duplicate records were identified in Zotero and manually reviewed before removal. The deduplicated records were then organized in a standardized Excel spreadsheet (Microsoft Corporation, Redmond, WA) for screening. Two review authors (AA and FME) independently screened titles and abstracts against the eligibility criteria. Articles considered potentially relevant by either review author were retrieved for full-text assessment, which was also completed independently by AA and FME. Disagreements were resolved through discussion, and unresolved cases were adjudicated by a third review author (AY). Full-text exclusion reasons were recorded study by study in the Excel spreadsheet using eligibility-based categories: wrong population, wrong imaging modality or exposure, wrong outcome or insufficient OCT/OCTA data, wrong study design or not original research, duplicate or overlapping population, and full text unavailable. No study was excluded because of low methodological quality or high risk of bias.

Data were extracted independently by two review authors using a standardized Excel extraction form. Extracted study-level information included first author, publication year, country, study design, disease group, participant characteristics, sample size, number of eyes where reported, comparator group, OCT or OCTA device, scan protocol, retinal layer or anatomical region assessed, reported outcomes, statistical format, and key findings. For quantitative synthesis, extracted data included the sample size, mean, and standard deviation for each eligible independent comparison group. The unit of analysis followed the reporting unit used by the source study, whether participants or eyes. For each outcome-specific meta-analysis, only one effect estimate per study was entered. When published eye-level summaries included both eyes from some participants, the data were used as reported; possible within-participant non-independence was recorded as a methodological concern and considered during risk-of-bias assessment and interpretation. When studies reported several related measurements within the same retinal domain, the most comparable whole-image, macular, parafoveal, peripapillary, or optic disc measure was selected according to the predefined outcome category to reduce duplication.

The primary outcomes were OCTA-derived superficial capillary plexus vessel density, deep capillary plexus vessel density, and radial peripapillary or optic disc vessel density. Secondary outcomes included FAZ area, FAZ morphology, RNFL or pRNFL thickness, GCC or GCL thickness, macular thickness, choroidal thickness, and other retinal microvascular metrics such as vascular fractal dimension, vascular orientation distribution, vessel diameter index, skeleton density, and perfusion density when reported. Outcomes were grouped by anatomical and imaging category before synthesis to improve comparability while acknowledging residual anatomical and methodological differences, including the grouping of radial peripapillary with optic disc vessel density measures and GCC with GCL thickness measures.

Risk of bias was assessed independently by two review authors after final study inclusion using design-appropriate Joanna Briggs Institute (JBI) critical appraisal checklists [11]. Analytical cross-sectional studies were assessed using the JBI checklist for analytical cross-sectional studies, case-control studies using the corresponding JBI case-control checklist, longitudinal cohort studies using the JBI cohort checklist [12], and non-randomized pre-post studies using the JBI quasi-experimental checklist [13]. Domain-level judgments considered participant selection, definition of disease and comparator groups, objective measurement of OCT or OCTA outcomes, control of confounding, completeness of data, and appropriateness of statistical analysis. Overall risk of bias was categorized as low, moderate, moderate-to-high, or high according to the number and seriousness of identified concerns. These judgments were used to contextualize individual study findings, help interpret heterogeneity, and temper conclusions from both pooled and narrative evidence; they were not used to determine study eligibility.

Continuous OCT- and OCTA-derived outcomes were synthesized using standardized mean differences with Hedges g when studies measured broadly comparable retinal domains but reported results using different scales, devices, scan sizes, segmentation boundaries, or measurement units. Hedges g expresses the between-group difference in pooled standard-deviation units; negative values indicated lower retinal vessel density or neuroretinal thickness measures in the ischemic stroke or carotid artery stenosis group than in the comparator group. Standardization was not assumed to make device-specific or anatomically distinct measurements fully interchangeable, and the pooled estimates were interpreted as research effect sizes rather than device-independent clinical thresholds. Random effects models were used for all meta-analyses because clinical and methodological heterogeneity was expected.

Separate meta-analyses were conducted for superficial capillary plexus or superficial vessel density, deep capillary plexus or deep vessel density, radial peripapillary or optic disc vessel density, FAZ area, RNFL or pRNFL thickness, and GCC or GCL thickness. Statistical heterogeneity was assessed using the I² statistic. Studies reporting odds ratios, regression results, correlation coefficients, subtype or contralateral-eye comparisons, or pre-post changes without an eligible independent control group were not pooled with continuous data from eligible independent comparison groups and were instead summarized narratively. Funnel plot asymmetry was not assessed because each outcome-specific meta-analysis included fewer than 10 studies.

Results

Study Selection

**Figure 1:**
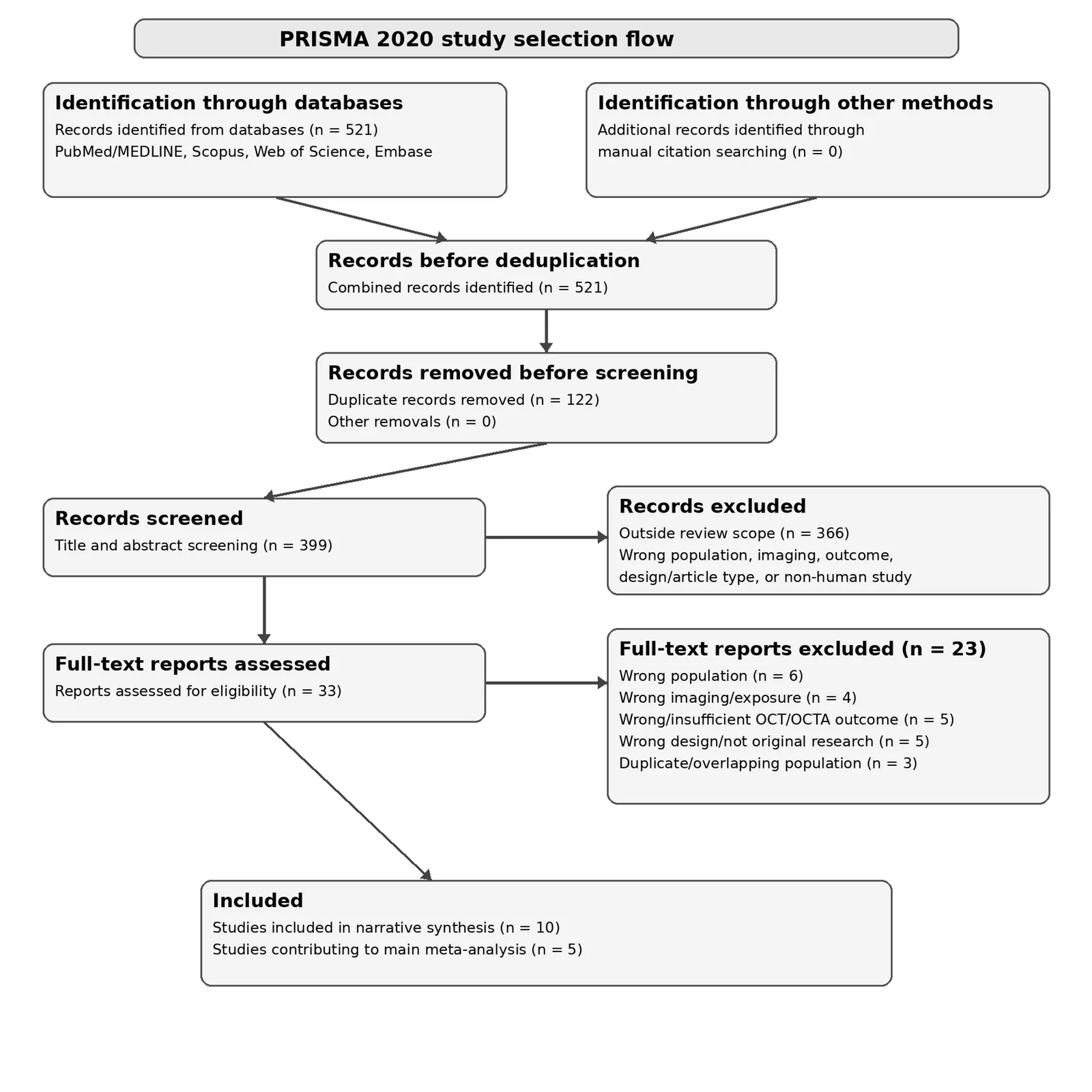
PRISMA 2020 study selection flow diagram. PRISMA: Preferred Reporting Items for Systematic Reviews and Meta-Analyses; OCT: optical coherence tomography; OCTA: optical coherence tomography angiography. Full-text reports were excluded only when they did not meet the predefined eligibility criteria.

Database searches identified 521 records, while manual citation searching did not identify any additional records. After 122 duplicate records were removed, 399 records were screened by title and abstract. Of these, 366 records were excluded because they were outside the review scope, including wrong population, wrong imaging modality or exposure, wrong outcome, wrong article type or design, or non-human or laboratory studies. A total of 33 full-text reports were assessed for eligibility, and 23 were excluded because they did not meet the predefined eligibility criteria: wrong population (n = 6), wrong exposure or imaging modality (n = 4), wrong outcome or insufficient OCT/OCTA data (n = 5), wrong study design or not original research (n = 5), and duplicate or overlapping population (n = 3). Ten original studies were included in the narrative synthesis, and five contributed data to the main continuous meta-analysis. The study-selection process is summarized in Figure 1.

Study Characteristics

The included studies were Duan et al. [14], Lahme et al. [15], Lee et al. [16], Li et al. [17], Liang et al. [18], Liu et al. [19], Liu et al. [20], Pierro et al. [21], Xu et al. [22], and Zhang et al. [23]. These studies evaluated OCT- or OCTA-derived retinal vascular and neuroretinal imaging measures in adults with ischemic stroke, ischemic stroke subtypes, carotid artery stenosis, or carotid atherosclerosis-related conditions. The main characteristics of the included studies are summarized in Table 3.

**Table 3:** Characteristics of included studies. CAS: carotid artery stenosis; CC: choriocapillaris; CIMT: carotid intima-media thickness; CTP: computed tomography perfusion; DCP: deep capillary plexus; DVC: deep vascular complex; FAZ: foveal avascular zone; FAR: FAZ axis ratio; FC: FAZ circularity; FD: fractal dimension; FR: FAZ roundness; GCL: ganglion cell layer; GCC: ganglion cell complex; ICP: intermediate capillary plexus; LAA: large-artery atherosclerosis; OCT: optical coherence tomography; OCTA: optical coherence tomography angiography; OR: odds ratio; PSM: propensity score matching; RPC: radial peripapillary capillary; RNFL: retinal nerve fiber layer; SAA: small-artery occlusion; SCP: superficial capillary plexus; SD: skeleton density; SD-OCT: spectral-domain optical coherence tomography; SMD: standardized mean difference; SS-OCTA: swept-source optical coherence tomography angiography; SVC: superficial vascular complex; SVP: superficial vascular plexus; VD: vessel density; VAD: vascular area density; VDI: vessel diameter index; VFD: vascular fractal dimension; VOD: vascular orientation distribution. The synthesis-decision column identifies whether each study contributed to the main continuous SMD meta-analysis or was summarized narratively because it reported association estimates, correlation coefficients, pre-post findings, or data that were not directly comparable with continuous outcomes from eligible independent comparison groups.

Study	Country	Design	Disease group	Disease sample	Comparator	Imaging	Main outcomes	Synthesis decision
Duan et al. [14]	China	Cross-sectional comparative study	Ischemic stroke; lacunar/nonlacunar subtypes	33 participants/52 eyes	27 controls/43 eyes	OCTA; AngioVue RTVue XR Avanti	VAD, VFD, VOD, FAZ area, FAR, FC, FR in SCP and DCP	Narrative: association estimates
Lahme et al. [15]	Germany	Prospective case-control plus pre/post follow-up	Severe asymptomatic carotid artery stenosis	25 CAS patients/eyes	25 healthy controls/eyes	OCTA; RTVue XR Avanti	Superficial/deep macular flow density, RPC flow density	Main SMD meta-analysis; pre-post findings summarized narratively
Lee et al. [16]	Taiwan	Retrospective pre/post interventional observational study	Severe carotid stenosis undergoing stenting	20 patients; ipsilateral and fellow eyes	No external healthy control	OCTA; XR Avanti AngioVue	SVC, DVC, RPC vessel density before and after CAS	Narrative: pre-post findings
Li et al. [17]	China	Prospective exploratory observational cohort with propensity score matching	Carotid artery stenosis	40 CAS recruited; matched eyes/patients after PSM	89 controls recruited; matched subset after PSM	AngioVue OCTA, Optovue	RPC, SVC, DVC, CC vessel density; RNFL; CTP agreement	Narrative: matched and pre-post data not directly comparable with the main meta-analysis
Liang et al. [18]	China	Cross-sectional retrospective comparative study	Ischemic stroke	159 ischemic stroke eyes/patients	109 age-matched controls/eyes	OCTA; RTVue-XR Avanti, Optovue	SCP, DCP, RPC VD; FAZ; GCC; RNFL	Main SMD meta-analysis
Liu et al. [19]	China	Prospective cross-sectional comparative study	Stroke confirmed by CT/MRI	189 stroke patients	195 controls	OCTA; AngioVue/Optovue platform	Macular SCP/DCP VD, FAZ metrics, FD-300, optic disc VD	Main SMD meta-analysis; overlap examined in sensitivity analysis
Liu et al. [20]	China	Prospective correlation study	Internal carotid artery stenosis	31 patients; 55 eyes	Contralateral eye comparison; no external healthy control	SS-OCTA	SVC, DVC, SVP, ICP, DCP; CTP hemodynamic parameters	Narrative: correlation coefficients
Pierro et al. [21]	Italy	Prospective observational case-control plus pre/post follow-up	Carotid artery stenosis	60 eyes of 30 CAS patients	30 eyes of 30 controls	OCT/OCTA	CT, RNFL, GCL, VD, vessel tortuosity	Main SMD meta-analysis; follow-up findings summarized narratively
Xu et al. [22]	China	Cross-sectional observational cohort	Carotid atherosclerosis/CIMT/plaque	138 participants; subgroups by CIMT/plaque	Normal CIMT/no-plaque groups	OCTA	VD, SD, VDI, FD, FAZ in superficial and deep retinal layers	Narrative: non-comparable carotid atherosclerosis subgroup data
Zhang et al. [23]	China	Prospective comparative stroke subtype study	Acute ischemic stroke subtypes	28 LAA and 41 SAA patients	65 age- and sex-matched controls	OCTA and SD-OCT	Retinal capillary VD and retinal layer thickness	Main SMD meta-analysis after pooling stroke subtypes

Key Findings

The key findings were organized by disease group and OCT/OCTA imaging measure domain. Studies that were not suitable for the main continuous meta-analysis were retained in the narrative synthesis when they reported association estimates, correlation coefficients, subtype comparisons, contralateral eye comparisons, or pre-post findings relevant to the review question. The study-level findings are summarized in Table 4.

**Table 4:** Key neurovascular findings from included studies. CAS: carotid artery stenosis; CT: computed tomography; DCP: deep capillary plexus; DVC: deep vascular complex; FAZ: foveal avascular zone; FAR: FAZ axis ratio; FC: FAZ circularity; GCC: ganglion cell complex; ONH: optic nerve head; OR: odds ratio; OCTA: optical coherence tomography angiography; pRNFL: peripapillary retinal nerve fiber layer; RNFL: retinal nerve fiber layer; RPC: radial peripapillary capillary; SCP: superficial capillary plexus; SMD: standardized mean difference; SVC: superficial vascular complex; VD: vessel density; VOD: vascular orientation distribution.

Study	Disease group	Key finding	Use in synthesis
Duan et al. [14]	Ischemic stroke	Higher DCP FAZ axis ratio and lower DCP FAZ circularity were independently associated with ischemic stroke. Lower SCP VOD was associated with lacunar infarction compared with nonlacunar infarction.	Narrative: association estimates
Lahme et al. [15]	Carotid artery stenosis	Severe CAS showed lower superficial macular and ONH/RPC flow density than controls, with ONH flow density improving after carotid endarterectomy.	Baseline independent-group SMD; pre-post findings summarized narratively
Lee et al. [16]	Carotid artery stenosis	Macular vessel density increased after carotid angioplasty and stenting, particularly in the deep vascular complex.	Narrative: pre-post findings
Li et al. [17]	Carotid artery stenosis	OCTA detected lower RPC, SVC, and DVC vessel density in carotid stenosis and postoperative improvement in ocular perfusion.	Narrative: matched and pre-post data
Liang et al. [18]	Ischemic stroke	Ischemic stroke patients had reduced SCP, DCP, and RPC vessel density, as well as reduced GCC and RNFL thickness. FAZ area was not significantly different.	Main SMD meta-analysis
Liu et al. [19]	Stroke	Stroke patients had reduced macular SCP and DCP vessel density, lower vessel density around the FAZ, and reduced optic disc vessel density after adjustment for vascular risk factors.	Main SMD meta-analysis; overlap examined in sensitivity analysis
Liu et al. [20]	Internal carotid artery stenosis	Superficial retinal microvascular perfusion correlated with CT perfusion-derived cerebral hemodynamic parameters.	Narrative: correlation coefficients
Pierro et al. [21]	Carotid artery stenosis	CAS was associated with thinner choroid and altered retinal vascular density or tortuosity, and retinal perfusion improved after endarterectomy.	Main SMD meta-analysis; follow-up findings summarized narratively
Xu et al. [22]	Carotid atherosclerosis	Macular vascular density, skeleton density, and fractal dimension decreased with increasing carotid atherosclerotic burden.	Narrative: non-comparable carotid atherosclerosis subgroup data
Zhang et al. [23]	Acute ischemic stroke subtypes	Deep retinal capillary vessel density was reduced in ischemic stroke subtypes compared with controls, and superior pRNFL differed across subtypes.	Main SMD meta-analysis after pooling stroke subtypes

Random-effects meta-analyses were conducted separately for each retinal vascular or neuroretinal structural outcome. Hedges g was used as the standardized mean difference. With the cerebrovascular disease group coded as the index group, negative Hedges g values indicated lower mean outcome values than in the eligible comparator groups. The pooled quantitative results are summarized in Table 5.

**Table 5:** Summary of random-effects meta-analysis results. CI: confidence interval; DCP: deep capillary plexus; FAZ: foveal avascular zone; GCC: ganglion cell complex; GCL: ganglion cell layer; I²: proportion of observed variability attributed to heterogeneity; pRNFL: peripapillary retinal nerve fiber layer; RNFL: retinal nerve fiber layer; RPC: radial peripapillary capillary; SCP: superficial capillary plexus. With the cerebrovascular disease group coded as the index group, negative Hedges g values indicate lower mean retinal vascular density or neuroretinal thickness values than in the comparator group. These estimates are standardized differences expressed in standard deviation units and should not be interpreted as device-independent clinical thresholds. Grouped outcomes represent broadly related retinal domains but retain anatomical and methodological differences.

Outcome	k comparisons	Pooled Hedges g	95% CI	I²	Interpretation
SCP/superficial vessel density	5	-0.329	-0.641 to -0.017	77.1%	Small pooled standardized difference; substantial heterogeneity
DCP/deep vessel density	5	-0.544	-0.778 to -0.311	58.4%	Moderate pooled standardized difference; moderate heterogeneity detected
RPC/optic disc vessel density	5	-0.590	-0.997 to -0.183	85.3%	Pooled standardized difference; high heterogeneity limits uniform interpretation
FAZ area	2	-0.229	-0.384 to -0.073	0%	Small exploratory standardized difference; 2 comparisons; no heterogeneity detected
RNFL/pRNFL thickness	3	-0.275	-0.467 to -0.083	0%	Small pooled standardized difference; 3 comparisons; no heterogeneity detected
GCC/GCL thickness	3	-0.329	-0.516 to -0.141	0%	Small pooled standardized difference; 3 comparisons; no heterogeneity detected

Outcome-Specific Forest Plots

**Figure 2:**
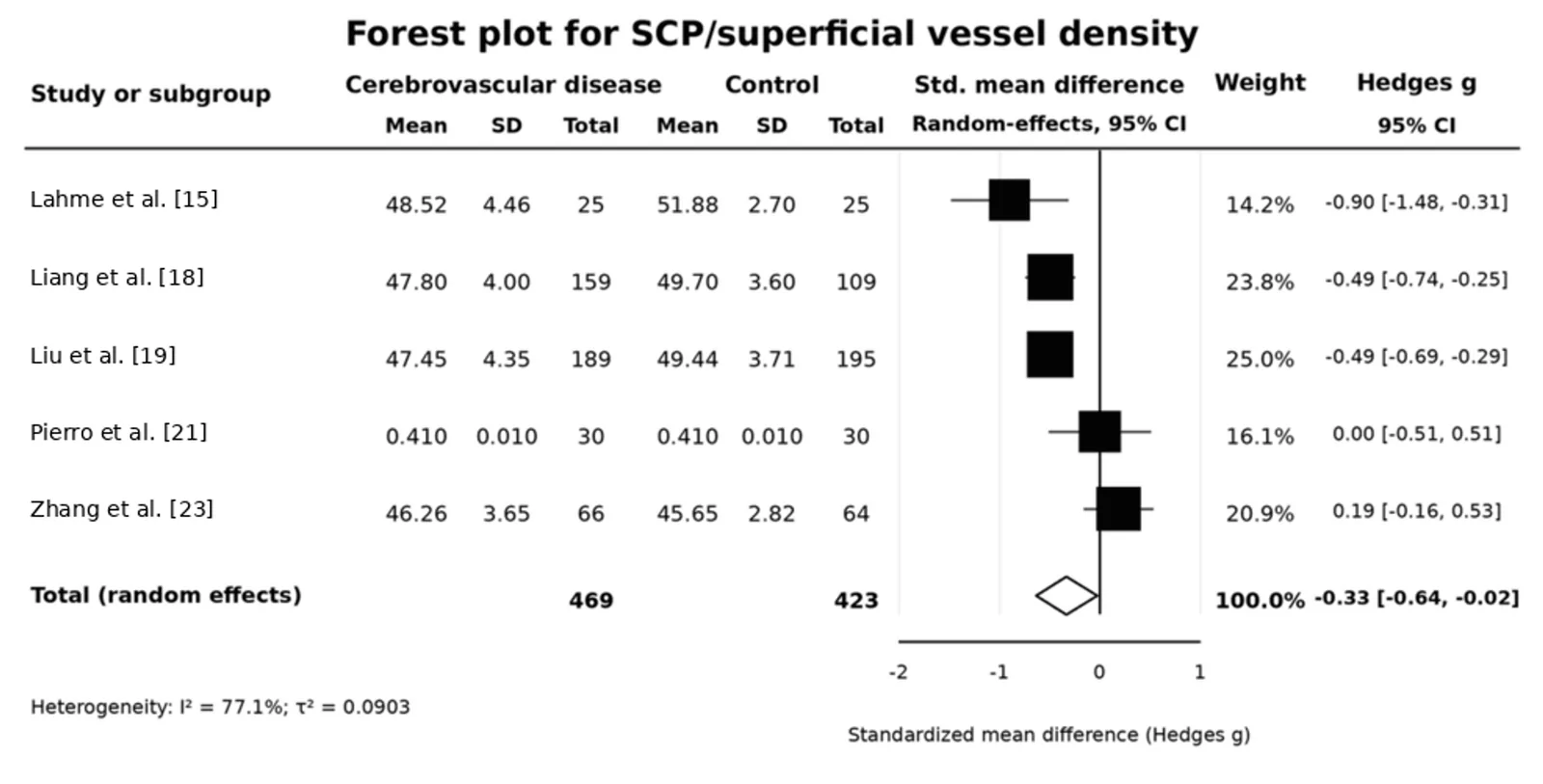
Forest plot for SCP/superficial vessel density. SCP: superficial capillary plexus; SMD: standardized mean difference; CI: confidence interval; SD: standard deviation. With the cerebrovascular disease group coded as the index group, negative Hedges g values indicate lower mean superficial vessel density values than in the comparator group.

Outcome-specific forest plots were generated for each pooled retinal vascular or neuroretinal structural outcome. Each plot reports the study name, reference number, index group and comparator group means and standard deviations, sample size, study weight, Hedges g, and the corresponding 95% confidence interval. Sample sizes represent participants or eyes according to the unit reported by each source study, as detailed in Table 3. The meta-analysis used the published aggregate data as reported. When bilateral eye-level observations were included, no additional within-participant clustering adjustment was applied at the meta-analysis level because individual-level data and within-person correlation estimates were unavailable; possible non-independence was considered during risk-of-bias assessment and interpretation. For superficial capillary plexus (SCP)/superficial vessel density, the analysis showed a small pooled standardized difference indicating lower values in the cerebrovascular disease group (Hedges g = -0.329, 95% CI: -0.641 to -0.017), with substantial between-study heterogeneity (I² = 77.1%). The magnitude of the difference therefore varied across the five comparisons. The forest plot is shown in Figure 2.

**Figure 3:**
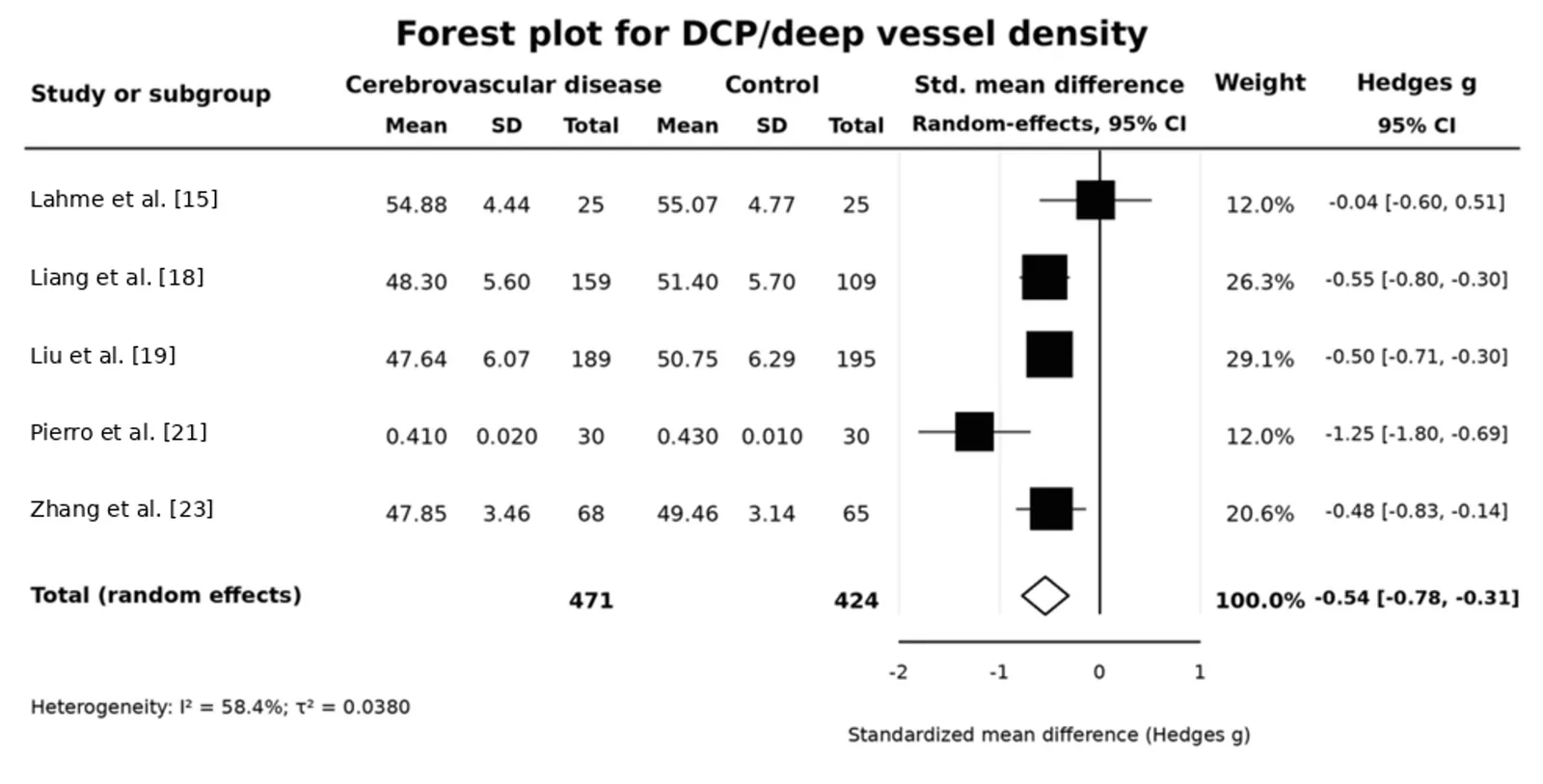
Forest plot for DCP/deep vessel density. DCP: deep capillary plexus; SMD: standardized mean difference; CI: confidence interval; SD: standard deviation. With the cerebrovascular disease group coded as the index group, negative Hedges g values indicate lower mean deep vessel density values than in the comparator group.

The deep capillary plexus (DCP)/deep vessel density analysis showed a moderate pooled standardized difference, with lower deep vessel density in the cerebrovascular disease group (Hedges g = -0.544, 95% CI: -0.778 to -0.311). Moderate heterogeneity was detected (I² = 58.4%) across the five comparisons. The forest plot is shown in Figure 3.

**Figure 4:**
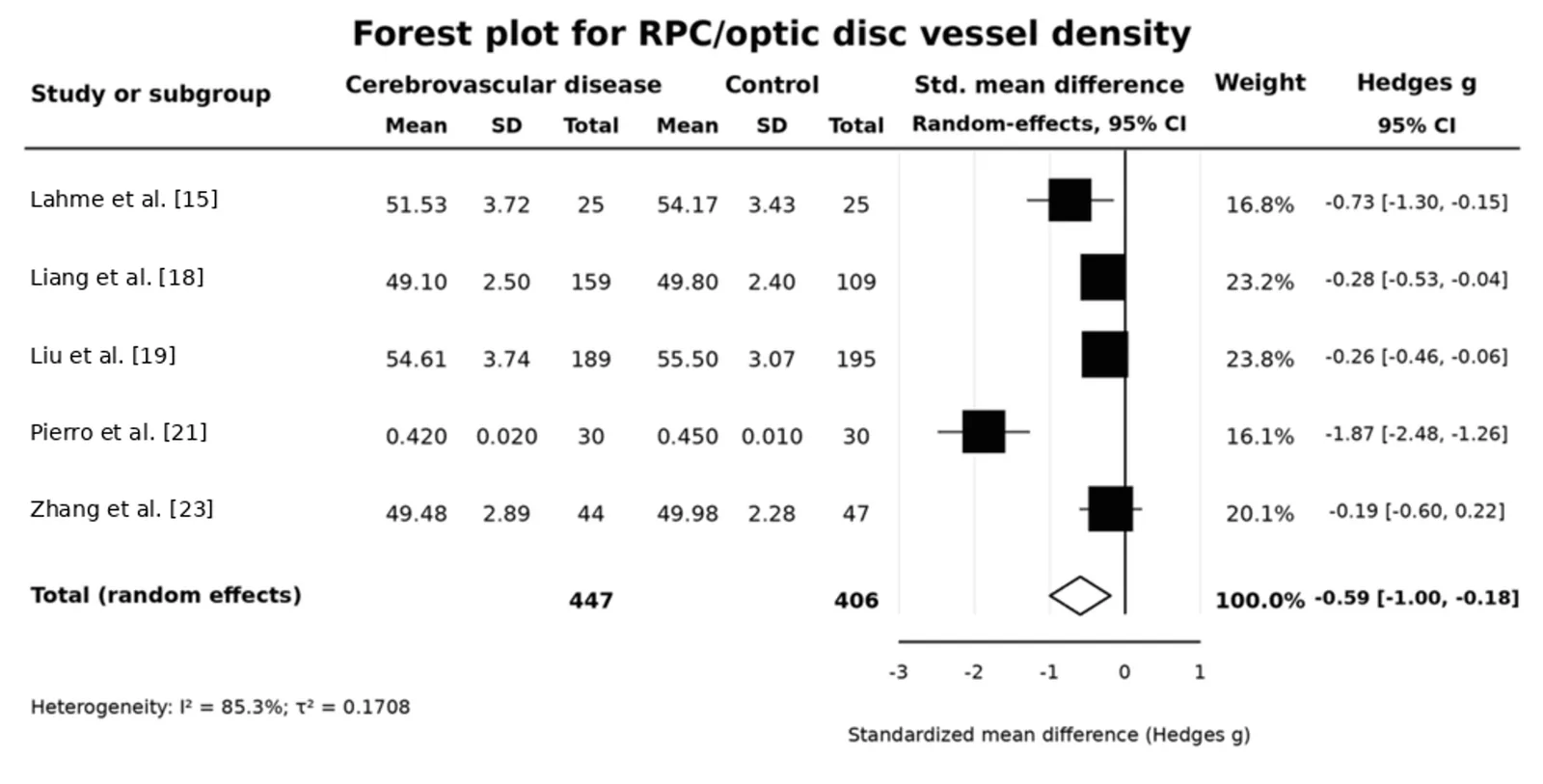
Forest plot for RPC/optic disc vessel density. RPC: radial peripapillary capillary; SMD: standardized mean difference; CI: confidence interval; SD: standard deviation. With the cerebrovascular disease group coded as the index group, negative Hedges g values indicate lower mean RPC or optic disc vessel density values than in the comparator group.

Radial peripapillary capillary (RPC) vessel density and optic disc vessel density were grouped as a broadly related peripapillary or optic nerve head vascular domain, while acknowledging that the contributing measurements may differ in anatomical coverage, device platform, segmentation boundaries, inclusion of large vessels, and metric definition. The analysis showed a pooled standardized difference indicating lower RPC or optic disc vessel density in the cerebrovascular disease group (Hedges g = -0.590, 95% CI: -0.997 to -0.183), although substantial between-study heterogeneity (I² = 85.3%) limits a uniform interpretation of the effect across the five comparisons. The forest plot is shown in Figure 4.

**Figure 5:**
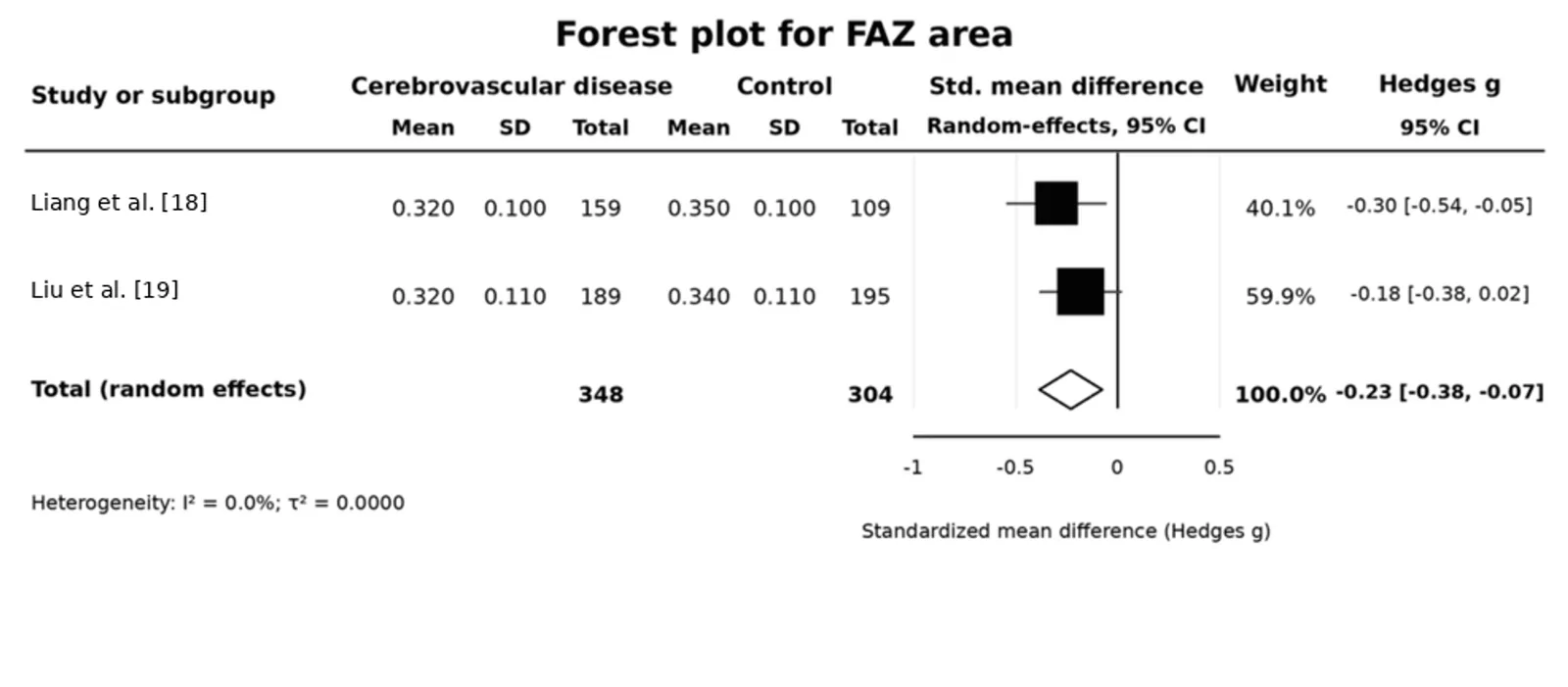
Forest plot for FAZ area. FAZ: foveal avascular zone; SMD: standardized mean difference; CI: confidence interval; SD: standard deviation. With the cerebrovascular disease group coded as the index group, negative Hedges g values indicate lower mean FAZ area values than in the comparator group.

The FAZ area analysis showed a small exploratory pooled standardized difference, with a lower mean FAZ area in the cerebrovascular disease group (Hedges g = -0.229, 95% CI: -0.384 to -0.073). Only two comparisons contributed to this analysis. No heterogeneity was detected (I² = 0%), but this estimate should be interpreted cautiously because of the small number of comparisons. The forest plot is shown in Figure 5.

**Figure 6:**
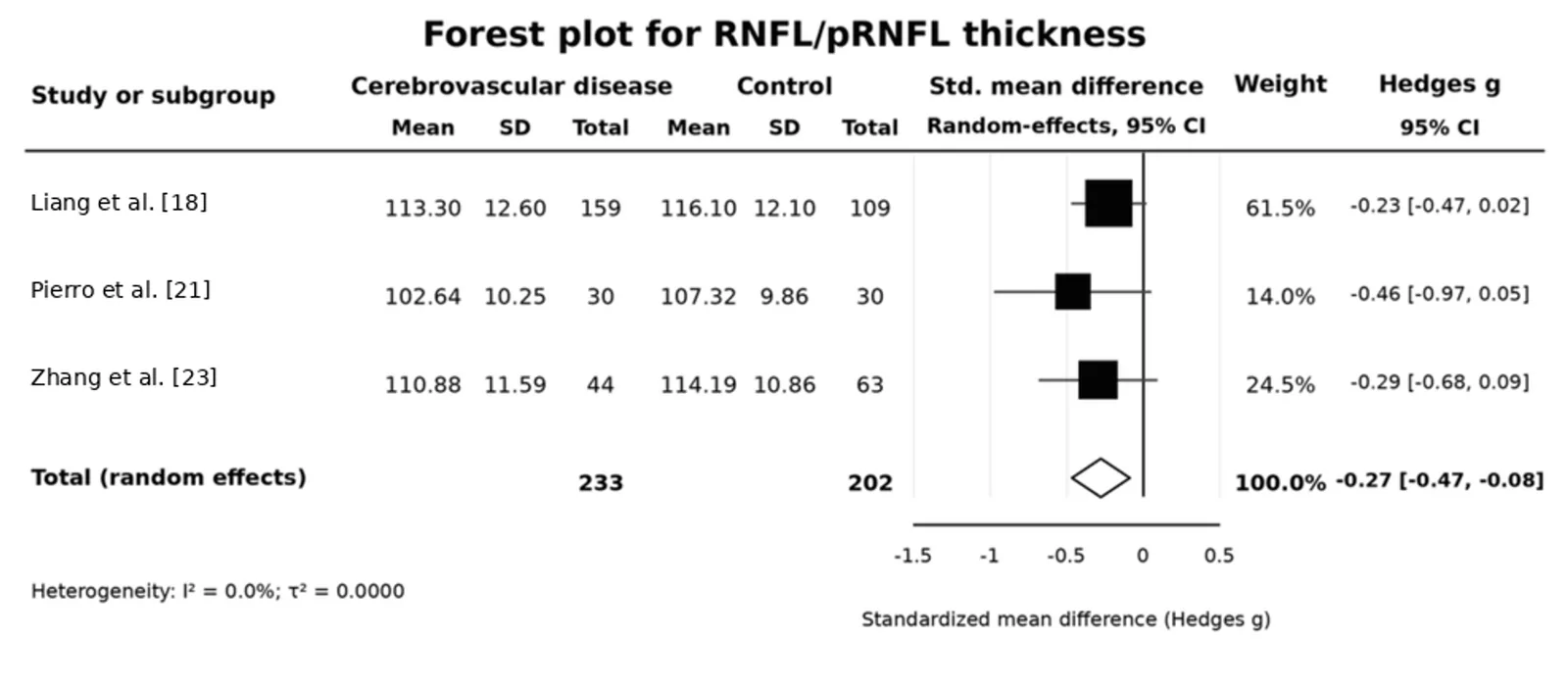
Forest plot for RNFL/pRNFL thickness. RNFL: retinal nerve fiber layer; pRNFL: peripapillary retinal nerve fiber layer; SMD: standardized mean difference; CI: confidence interval; SD: standard deviation. With the cerebrovascular disease group coded as the index group, negative Hedges g values indicate lower mean RNFL or pRNFL thickness values than in the comparator group.

The RNFL/pRNFL thickness analysis showed a small pooled standardized difference indicating lower RNFL or pRNFL thickness in the cerebrovascular disease group (Hedges g = -0.275, 95% CI: -0.467 to -0.083). The estimate was based on three comparisons and combined related but anatomically distinct RNFL and pRNFL measurements. No heterogeneity was detected (I² = 0%), although the small number of comparisons limits the strength of this inference. The forest plot is shown in Figure 6.

**Figure 7:**
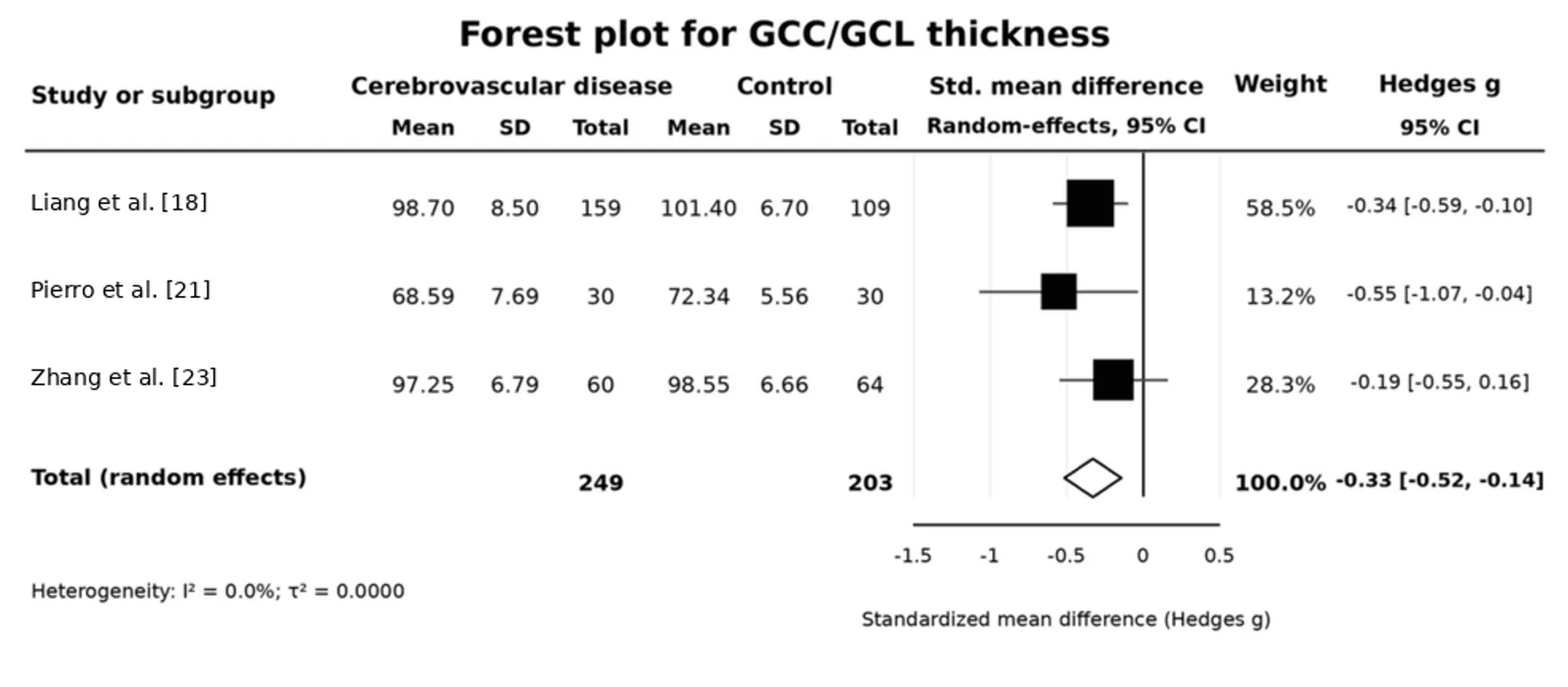
Forest plot for GCC/GCL thickness. GCC: ganglion cell complex; GCL: ganglion cell layer; SMD: standardized mean difference; CI: confidence interval; SD: standard deviation. With the cerebrovascular disease group coded as the index group, negative Hedges g values indicate lower mean GCC or GCL thickness values than in the comparator group.

The GCC/GCL thickness analysis showed a small pooled standardized difference indicating lower GCC or GCL thickness in the cerebrovascular disease group (Hedges g = -0.329, 95% CI: -0.516 to -0.141). The estimate was based on three comparisons and combined related but anatomically and methodologically distinct GCC and GCL measurements. No heterogeneity was detected (I² = 0%), but this does not establish true homogeneity because the analysis included only three comparisons. The forest plot is shown in Figure 7.

Risk of Bias

Risk-of-bias findings were summarized across adapted domains derived from the relevant design-specific JBI checklist items, including selection, comparability and confounding, disease ascertainment, outcome measurement, missing data, and statistical analysis. The appraisal was completed after study inclusion and was used to contextualize the pooled and narrative findings rather than to determine eligibility. The study-level judgments are summarized in Table 6.

**Table 6:** Risk-of-bias assessment of included observational studies. ROB: risk of bias; CAS: carotid artery stenosis; PSM: propensity score matching; SMD: standardized mean difference. The table presents adapted domain-level summaries derived from the relevant design-specific Joanna Briggs Institute (JBI) checklist items. Domain-level judgments were categorized as low concern, some concerns, or high concern. Overall risk of bias was categorized as low, moderate, moderate-to-high, or high according to the number and seriousness of study-level limitations. Risk of bias was not used as an eligibility criterion.

Study	Selection	Comparability/confounding	Disease ascertainment	Outcome measurement	Missing data	Statistical analysis	Overall ROB	Main rationale
Duan et al. [14]	Some concerns	High concern	Low concern	Low concern	Some concerns	Some concerns	Moderate to high	Small sample, baseline imbalance, and possible non-independence from including both eyes
Lahme et al. [15]	Some concerns	Some concerns	Low concern	Low concern	Some concerns	Some concerns	Moderate	Prospective design with controls, but small sample and vascular comorbidity differences
Lee et al. [16]	Some concerns	High concern	Low concern	Low concern	Some concerns	Some concerns	Moderate to high	Pre/post design without independent healthy controls; not suitable for main SMD
Li et al. [17]	Some concerns	Some concerns	Low concern	Low concern	Some concerns	Some concerns	Moderate	Prospective cohort with PSM but converted data and complex eye-level comparisons require caution
Liang et al. [18]	Some concerns	Some concerns	Low concern	Low concern	Some concerns	Low concern	Moderate	Baseline imbalance adjusted statistically; retrospective cross-sectional design
Liu et al. [19]	Some concerns	Some concerns	Low concern	Low concern	Some concerns	Low concern	Moderate	Large sample and adjustment, but cross-sectional design and possible overlap with later cohort
Liu et al. [20]	Some concerns	Some concerns	Low concern	Low concern	Some concerns	Some concerns	Moderate	Small sample and correlation design without external healthy control
Pierro et al. [21]	Some concerns	Some concerns	Low concern	Low concern	Some concerns	Some concerns	Moderate	Prospective design but limited sample and systemic risk-factor confounding
Xu et al. [22]	Some concerns	Some concerns	Low concern	Low concern	Some concerns	Some concerns	Moderate	Cross-sectional carotid atherosclerosis cohort; secondary relevance to clinical CAS/stroke
Zhang et al. [23]	Some concerns	Some concerns	Low concern	Low concern	Some concerns	Some concerns	Moderate	Stroke subtype study with controls, but subgroup pooling and sample size limitations require caution

Discussion

Principal Findings

This systematic review and meta-analysis identified pooled group-level differences in selected retinal microvascular and neuroretinal measurements among adults with ischemic stroke or carotid artery stenosis. Across eligible comparisons, average superficial vessel density, deep vessel density, radial peripapillary or optic disc vessel density, RNFL or pRNFL thickness, and GCC or GCL thickness were lower in the cerebrovascular disease groups than in comparator groups. The magnitude, precision, and between-study heterogeneity of the estimates varied across outcomes, populations, and imaging protocols. Because Hedges g expresses differences in standard-deviation units, these estimates should not be interpreted as direct measures of anatomical damage or clinical severity.

Comparison of Stroke and Carotid Stenosis Findings

The ischemic stroke studies provided broadly concordant but methodologically heterogeneous signals of retinal microvascular alteration. Liang et al. [18] and Liu et al. [19] reported lower superficial and deep macular vessel density measurements in patients with stroke, while Zhang et al. [23] reported lower deep retinal capillary vessel density across acute ischemic stroke subtype comparisons. Duan et al. [14] reported separate associations involving FAZ morphology and vascular orientation. Because these studies evaluated different retinal constructs and some used association or subtype analyses rather than directly comparable continuous outcomes, they do not demonstrate uniform or quantitatively consistent effects across the ischemic stroke literature.

Carotid artery stenosis studies addressed a related vascular condition rather than established stroke alone. Lahme et al. [15] and Pierro et al. [21] reported lower retinal or optic nerve head perfusion measurements in carotid artery stenosis, while Li et al. [17] reported lower retinal vessel density together with cerebral perfusion assessment. Lee et al. [16], Lahme et al. [15], and Pierro et al. [21] also reported changes in selected retinal perfusion parameters after carotid revascularization. These pre- and post-findings suggest that selected retinal perfusion measures may change after carotid revascularization. However, the absence of randomized comparison groups and differences in study design and comparator structure limit any causal interpretation. These studies were therefore summarized narratively rather than pooled with the continuous data from eligible independent comparison groups.

Retinal Microvascular Density Findings

The pooled analysis showed a small standardized difference toward lower superficial vessel density, but between-study heterogeneity was substantial. This variation may reflect differences in disease stage, stroke subtype, carotid stenosis severity, scan protocol, device platform, segmentation method, and vascular risk profile. Liang et al. [18] and Liu et al. [19] reported lower superficial vessel density measurements in stroke, whereas Lahme et al. [15] and Pierro et al. [21] reported lower superficial perfusion measurements in carotid stenosis. The pooled estimate should therefore be interpreted cautiously because it combined clinically distinct populations and methodologically varied imaging assessments.

The pooled analysis showed lower deep vessel density in the cerebrovascular disease groups, although moderate heterogeneity remained across the included comparisons. Liang et al. [18], Liu et al. [19], and Zhang et al. [23] reported lower deep retinal vascular measurements in ischemic stroke populations, while Duan et al. [14] reported separate associations involving FAZ morphology. These findings support possible deep retinal microvascular involvement, but they do not establish that deep plexus measures are more sensitive to ischemic injury than other retinal parameters.

The pooled analysis also showed lower average radial peripapillary or optic disc vessel density measurements. Liang et al. [18] reported lower radial peripapillary capillary density in ischemic stroke, while Lahme et al. [15] and Li et al. [17] reported lower optic nerve head or radial peripapillary perfusion measurements in carotid stenosis. However, radial peripapillary and optic disc measures are anatomically related but not directly equivalent, and heterogeneity was high. Differences in device type, scan size, segmentation, and inclusion of large vessels further limit a uniform interpretation of the pooled estimate.

Neuroretinal and FAZ Findings

Selected neuroretinal thickness measures were lower on average in cerebrovascular disease groups than in comparator groups. Liang et al. [18] reported lower GCC and RNFL thickness in ischemic stroke, while Zhang et al. [23] reported differences in peripapillary RNFL across acute ischemic stroke subtypes. Pierro et al. [21] reported structural and choroidal findings in carotid stenosis, although the more prominent findings involved choroidal and vascular measures. The pooled RNFL or pRNFL and GCC or GCL analyses showed small standardized differences, with no heterogeneity detected; however, only three comparisons contributed to each analysis, and the combination of RNFL with pRNFL and GCC with GCL restricts conclusions about consistency.

FAZ area and morphology were evaluated using limited and methodologically different evidence. Liang et al. [18] found no statistically significant FAZ area difference in an individual ischemic stroke comparison, whereas the pooled FAZ area analysis showed a small standardized difference based on only two comparisons. Duan et al. [14] reported exploratory associations involving FAZ axis ratio and circularity, but these association estimates are not directly comparable with pooled continuous effect sizes. FAZ measurements may also vary with image processing and segmentation. FAZ area and morphology may provide complementary information, but the current evidence does not establish that shape parameters outperform FAZ area or support a demonstrated hierarchy between these measures.

Relationship With Cerebral Hemodynamics and Prior Literature

Several studies examined relationships between retinal OCTA measurements and broader cerebrovascular physiology. Li et al. [17] reported retinal perfusion findings alongside cerebral perfusion assessment in carotid stenosis. Liu et al. [20] found correlations between superficial retinal perfusion and CT perfusion-derived cerebral hemodynamic parameters in internal carotid artery stenosis. Xu et al. [22] reported associations between retinal microvascular metrics and carotid atherosclerotic markers, including carotid intima-media thickness and plaque. These observational associations provide contextual support for a relationship between retinal measurements and cerebrovascular or carotid vascular features, but they do not establish diagnostic or prognostic performance.

These findings can also be considered alongside earlier literature on conventional retinal vascular signs and stroke risk. Yatsuya et al. [7] reported associations between retinal microvascular abnormalities and lacunar stroke risk, while McGeechan et al. [8] linked retinal vessel caliber with incident stroke events. Girach et al. [9] summarized evidence relating retinal imaging features to stroke risk and noted that OCT-based measures were less extensively studied than conventional fundus-derived signs. The present review extends this background by focusing specifically on OCT- and OCTA-derived retinal microvascular and neuroretinal measurements in adults with established ischemic stroke or carotid artery stenosis.

Clinical and Research Implications

The current evidence supports OCT and OCTA as potentially useful research tools for investigating retinal changes associated with cerebrovascular disease. It is not sufficient to support OCT or OCTA as standalone screening, diagnostic, prognostic, or risk-stratification tools for ischemic stroke or carotid artery stenosis. Most included studies were observational, comparator structures varied, and acquisition and segmentation methods were not standardized. These measurements should therefore be interpreted as exploratory and complementary research outcomes rather than established clinical decision measures.

Future studies should use prospective designs, standardized OCT and OCTA acquisition protocols, consistent segmentation and outcome definitions, and adequate adjustment for vascular and ocular confounders. Clear reporting of participant-level and eye-level data, including sample size, mean, and standard deviation for each outcome, would improve future quantitative syntheses. FAZ morphology, vascular orientation, and retinal vascular complexity warrant further evaluation as complementary outcomes, with prespecified acquisition and analysis methods.

Strengths and Limitations

Strengths of this review include a transparent eligibility and study selection process, outcome-specific quantitative pooling, explicit narrative synthesis of evidence that could not be meaningfully combined, and preservation of relevant observational evidence. Ischemic stroke and carotid artery stenosis were distinguished in the narrative synthesis, and design-appropriate risk-of-bias assessments were used to inform interpretation.

Several limitations should be acknowledged. The protocol was not prospectively registered, which limits external verification of prespecified methods. The evidence base was predominantly observational, and several studies had moderate or higher risk-of-bias concerns. Most pooled outcomes included only a small number of comparisons, limiting precision, sensitivity analyses, and assessment of small-study effects. Some quantitative analyses combined ischemic stroke and carotid artery stenosis populations and grouped related but non-identical measurements, including radial peripapillary with optic disc vessel density, RNFL with pRNFL, and GCC with GCL. Heterogeneity was substantial for superficial vessel density and high for radial peripapillary or optic disc vessel density. Differences in devices, scan sizes, segmentation methods, eye selection approaches, comparator structures, and confounder adjustment further limited direct comparability. Some source studies reported eye-level data, and the available aggregate data did not permit further adjustment for within-participant correlation. Cross-sectional studies could not determine temporality, while regression, correlation, and pre- and post-revascularization studies could not be included in the main standardized mean difference analyses.

Taken together, selected retinal vessel density and neuroretinal thickness measurements were lower on average in adults with ischemic stroke or carotid artery stenosis than in eligible comparator groups. However, the magnitude and generalizability of these group-level differences remain uncertain because of sparse comparisons, mixed populations, variable imaging methods, risk-of-bias concerns, and between-study heterogeneity. OCT and OCTA should therefore be considered research tools for studying retinal changes associated with cerebrovascular disease rather than validated pathological, diagnostic, or prognostic measures.

## Conclusions

This systematic review and meta-analysis identified lower average retinal vessel density and neuroretinal thickness measurements in adults with ischemic stroke or carotid artery stenosis compared with eligible comparator groups. These findings support OCT and OCTA as potentially useful research tools for investigating retinal changes associated with cerebrovascular disease.

The findings should be interpreted cautiously because the evidence was predominantly observational, several pooled outcomes included only a small number of comparisons, and substantial variation existed in populations, devices, scan protocols, segmentation methods, and comparator structures. Prospective studies with standardized acquisition methods, consistent outcome definitions, and adequate adjustment for vascular and ocular confounders are required before these imaging measures can be evaluated for diagnostic, prognostic, or risk-stratification use.
